# Advancing Cultural Competency in Allied Health Education: A Narrative Review

**DOI:** 10.7759/cureus.95590

**Published:** 2025-10-28

**Authors:** Grace A Valdez

**Affiliations:** 1 Allied Health and Kinesiology, Hofstra University, Hempstead, USA

**Keywords:** allied health education, cultural competency, curriculum implementation, diversity education, health disparities

## Abstract

As the U.S. population becomes increasingly diverse, allied health care providers must be equipped to deliver culturally competent and equitable care. While nursing education has long emphasized cultural competence, its integration into allied health disciplines varies. This narrative review synthesizes evidence from 2015 to 2025, examining three main approaches to cultural competence training in allied health education: cultural immersion, didactic coursework, and workshops or simulations. The findings suggest that no single strategy is sufficient. Cultural immersion promotes self-awareness and empathy through direct engagement with diverse communities; however, these experiences can be resource-intensive and hard to sustain. Didactic coursework offers a necessary theoretical foundation but can remain too abstract unless paired with practical experiences. Workshops and simulation-based training improve communication and clinical skills, but their benefits often decline without ongoing reinforcement. A hybrid approach, combining coursework, immersion, and repeated practice via workshops or simulations, produces the most consistent and lasting improvements. Reflection also plays a key role in reinforcing skill and attitude retention. Barriers to implementation include varying faculty readiness, limited access to immersion experiences, and reliance on self-reported outcomes. Specific fields such as radiologic sciences, speech-language pathology, dietetics, and respiratory therapy are underrepresented in the literature. Emerging methods, including interprofessional learning, virtual simulation, and telehealth-based cultural encounters, present promising pathways for scalable training. Overall, the evidence supports integrating cultural competence throughout allied health curricula over time, rather than treating it as a standalone requirement. Continuous, integrated approaches better prepare students to deliver equitable, patient-centered care across diverse clinical settings.

## Introduction and background

There has been a growing diversity in the U.S. population, leading to increased demand for culturally sensitive healthcare. Cultural and linguistic beliefs, along with economic conditions, significantly influence patients’ health behaviors, outcomes, and compliance with providers [1,2]. Ongoing inequities, especially among marginalized groups, highlight systemic injustices and the effects of care delivered without cultural inclusion. Therefore, it is increasingly recognized that cultural competence has become essential in providing high-quality care and is a professional obligation across all health disciplines.

Historically, nursing education has been a leader in integrating cultural competence into curricula, although allied health programs show similar efforts with notable differences [3]. Research indicates that while allied health students may acquire knowledge, they often lack the confidence to apply it in practice; Volberding found that athletic training students with higher cultural competence scores also reported greater confidence in providing culturally competent care [4]. This uneven emphasis creates gaps in preparing graduate students to deliver equitable care to diverse populations. Cultural training in most allied health programs is usually offered as optional workshops or short modules rather than being fully integrated into the entire curriculum. As a result, graduates might have strong technical and clinical skills but may feel less confident in communicating effectively with patients from different cultural or socioeconomic backgrounds.

The process of cultural competence, developed by Campinha-Bacote, and Purnell's model for cultural competence are two theoretical frameworks that provide essential paradigms for understanding this process. Both emphasize that competence is not a one-time achievement but an ongoing journey of learning, practicing, and self-questioning - about how to handle specific situations and cultural differences. These models recognize that competence goes beyond knowledge; it involves humility, adaptability, and a willingness to examine one's own biases. Without this mindset, cultural training can become just a method of "learning facts" rather than meaningful professional growth [1,2].

Research shows that organized cultural competence interventions are effective. Learners who receive cultural awareness training continue to respond positively in communication, patient relationships, and personal confidence. These improvements are seen across various types of interventions, such as cultural immersion, didactic coursework, and training workshops. However, studies also indicate that even in-depth interventions or single experiences tend to have low long-term retention, as skills often fade without ongoing practice. Therefore, it suggests that cultural competence is best developed through a hybrid approach over time, layering different strategies throughout a student’s entire educational journey.

It is important to identify which training approaches are most effective and how to promote allied health training efforts. A narrative review of the literature also provides best practices, challenges, and innovations in cultural competence education. Unlike earlier reviews that focused mainly on nursing or medicine, this paper concentrates exclusively on allied health education, highlighting newer interventions and emerging fields that have been less covered in the literature. By comparing the outcomes of immersion experiences, classroom-based education, and workshop training, this paper aims to determine which strategies best "teach" allied health students for practice in a complex real-world clinical environment.

Cultural competence in medicine has been studied for decades, and several models assist in developing this skill. Both Campinha-Bacote’s Process of Cultural Competence and Purnell’s Model for Cultural Competence highlight that cultural competence is an ongoing process, not a final goal. It requires continuous growth in knowledge, skills, and self-awareness, along with multiple interactions with diverse populations [1,2]. These models remind us that cultural competence is dynamic, rooted in both formal education and lived experience.

Although well-known, cultural competence has not been consistently incorporated into allied health education. In nursing, it is common to include cultural competence as a requirement in the curriculum, but teaching it varies greatly in athletic training, physical therapy, occupational therapy, and radiologic sciences. This uneven focus leads to gaps in readiness, resulting in well-trained students with strong clinical skills but insufficient confidence to work with patients from diverse cultural backgrounds.

Interventions in education, when well-structured, can have a meaningful impact. Students participating in cultural immersion, classroom-based education, or skills-focused workshops report improvements in communication, cultural sensitivity, and clinical confidence [5,6]. Recently, new educational tools such as simulation-based patient encounters, interprofessional learning modules, and telehealth cultural exchanges have been introduced. These findings suggest that cultural competence is probably best achieved through a blend of approaches rather than relying on method-specific training.

At the same time, obstacles to mainstreaming persist. The faculty's readiness to teach cultural concepts varies, and the curriculum is already rich with technical and clinical content. Programs may face challenges in securing clinical placements that reflect diverse patient populations or in maintaining relationships with communities that facilitate immersion experiences. There are also financial and logistical barriers that restrict access to international or community-based training opportunities. These issues highlight the need for culturally responsive education to be both evidence-based and practical, sustainable, and applicable across various allied health settings.

## Review

Methods

A review was structured through a search of peer-reviewed literature. The focus was on studies published in the last 10 years (2015-2025) that investigated cultural competence education in allied health programs. Older articles referencing frameworks and theoretical contexts were included. The databases searched included PubMed, CINAHL, Scopus, and Google Scholar. Search terms combined relevant keywords such as cultural competence, cultural humility, cultural immersion, diversity training, workshops, didactic education, and allied health education. Boolean search strings combined terms like (“cultural competence” OR “cultural humility”) AND (“allied health” OR “health professions”). Searches were limited to peer-reviewed, English-language studies published from 2015 to 2025. After removing duplicates, approximately 125 records were screened by title and abstract, with 25 meeting the inclusion criteria after full-text review. The goal was to identify studies examining cultural competence training through immersion experiences, didactic coursework, or workshop- or simulation-based education within allied health programs.

Inclusion criteria included articles (1) published in English; (2) involving students and/or professionals in allied health professions such as athletic training, physical therapy, occupational therapy, speech-language pathology, radiologic sciences, pharmacy, or related fields; and (3) assessing at least one of the three main strategies: immersion experiences, didactic education, or workshops with measurable outcomes, including cultural competency, competence, skill, or knowledge. Exclusion criteria included studies focused solely on medical or nursing students (without comparison to allied health), opinion pieces, non-data-based articles, and grey literature.

The final group of studies included a mix of qualitative, quantitative, and mixed-methods approaches, along with several systematic and scoping reviews. This provided a balance between breadth and depth of evidence-covering outcome measures as well as narrative accounts of student experiences and implementation challenges. By focusing the review on allied health care and these three strategies, we aimed to identify common themes, highlight best practices, and outline barriers in the field.

Review

There is still uneven cultural competency across the U.S. healthcare system, and reports often highlight the consequences in terms of misdiagnoses, misunderstandings, and disparities. This issue is significant for allied health professionals to work effectively across cultures, languages, and socioeconomic levels. While cultural competence training is a standard part of nursing curricula, allied health programs vary in how consistently they include it. Core models, namely, Campinha-Bacote’s Process of Cultural Competence and Purnell’s Model for Cultural Competence, view competence as an ongoing journey that requires self-reflection, skill development, and repeated engagement with diverse populations. Students involved in structured cultural training programs consistently report improved communication, greater confidence, and increased awareness. Please see Table 1 for an overview of the reviewed studies.

**Table 1:** Summary of key studies on cultural competence education in allied health

Author(s), year	Discipline(s)	Intervention type	Outcomes	Limitations / notes
Knecht et al., 2019 [5]	Healthcare students	10-week cross-cultural service-learning program	Higher IAPCC-SV scores; increased confidence in diverse interactions	Single-site study, small sample size
Almutairi et al., 2017 [6]	Nursing	Clinical exposure with diverse patient populations	Direct patient encounters most influential for competence	Single cultural context; limited generalizability
DiBiasio et al., 2023 [7]	Physical therapy	Multi-site intercultural learning experiences (n ≈ 1,000)	Higher cultural competence scores in exposed students	Strong design, but limited to PT programs
Ceo-DiFrancesco et al., 2022 [8]	Occupational therapy	Two-week immersion in Guatemala with reflection	Pre/post competence gains; transformative shifts	Short duration; retention unclear
Chae et al., 2023 [9]	Nursing	Immersive VR simulations	VR was engaging and realistic to support experiential learning	Small sample size; no longitudinal assessments
Hunter et al., 2025 [10]	Health profession educators	VR simulation modules	Students reported greater confidence and readiness for clinical practice	Small sample size; self-reported outcomes only
Singh et al., 2023 [11]	Speech-language pathology, occupational therapy, physiotherapy, dietetics	Qualitative interpretive description study of lived clinical practice	Increased awareness of their biases, enhanced ethical sensitivity, and illuminated tensions between patient/family values and institutional constraints	Small sample size, limited diversity in provider demographics
Cáceres-Titos et al., 2025 [12]	Palliative and end-of-life care	Interviews/focus groups; immersive clinical context study	Fostered humility and awareness but also highlighted ethical dilemmas	Limited generalizability and no long-term outcome measures
Brentnall et al., 2024 [13]	Physiotherapy, occupation therapy, speech pathology, dietetics	Interprofessional immersive simulation program	Increased preparedness, confidence, and understanding of teamwork and patient-centered care	Self-report measures may limit generalizability
Stough-Hunter et al., 2016 [14]	Health sciences students	Interactive conversations, case-based learning, lectures	Interactive/case-based > lecture for awareness & communication	No long-term follow-up
Fryer et al., 2020 [15]	Allied health students	Four-week cultural competency module	Improved knowledge/awareness; limited clinical confidence	Didactic alone insufficient
Arruzza & Chau, 2021 [16]	Multiple health professions	Scoping review of classroom interventions	Consistent improvements in knowledge, skills, attitudes	Methodological limitations across studies
Ozcan Edeer & Rust, 2022 [17]	PT and OT	Longitudinal interprofessional curriculum	Increased awareness, skills, knowledge	Resource-intensive
O’Fallon & Garcia, 2023 [18]	Speech-language pathology and audiology	Integration of active learning strategies	Active learning increased cultural awareness, reflection, and application	Mostly descriptive results lacked quantitative outcomes
Fernández-Alcántara et al., 2025 [19]	Health professions	Literature review of virtual simulation tools	Enhanced communication skills, empathy and learner engagement	Limited long-term evaluation and inconsistent outcome measures.
Abrishami, 2023 [20]	Radiologic technology	Cultural units within curriculum	Modest empathy improvements; call for deeper integration	Minimal exposure, limited impact
Chen et al., 2015 [21]	Pharmacy (PharmD)	HL/CC workshops	Immediate knowledge gains; skills declined over time	No reinforcement; short-term effect
Jones & Pinto-Zapp, 2017 [22]	Allied health students	Cross-cultural training workshops	Increased awareness of bias; skills eroded	Limited scope, short-term
Kempny et al., 2025 [23]	Mixed health professions	Online diversity training (scoping review)	Knowledge and self-efficacy improved; bias confronted	Few long-term outcomes measured
Walkowska et al., 2023 [24]	Health professions students	Simulated patient encounters (27 studies)	Greater competence, confidence, satisfaction	Variation in simulation quality
Walshe et al., 2022 [25]	Healthcare education	Review of cultural simulations	Effective; ethical design critical	Risk of stereotyping if poorly designed

Cultural Immersion Experiences

One of the most effective ways to develop competence is through immersion. By engaging in different communities both inside and outside of D.C., students are encouraged to participate, utilize their skills, and communicate within the context of a new culture. Knecht et al. found that this had a tangible effect: participants in a 10-week cross-cultural service-learning course scored significantly higher on the IAPCC-SV and reported greater confidence compared to non-participants [5]. Almutairi et al. also confirmed that applying learned content - rather than just classroom instruction - was the most influential factor on confidence [6]. Local service-learning programs that are well-designed may also be effective, even if foreign placements are not possible due to financial or logistical challenges.

An example of evidence on a larger scale can be seen in DiBiasio et al., who surveyed eight U.S. PT programs, involving approximately 1,000 students [7]. Their multi-institutional study showed that intercultural experiences were a strong predictor of higher competence scores, confirming that the benefits persisted across different institutions. Ceo-DiFrancesco et al. described a two-week OT immersion in Guatemala [8]. Although brief, adding structured reflection time helped students turn cultural dissonance into empathy and understanding.

In addition to traditional placements, new work is exploring digital or hybrid immersion. Chae et al. tested immersive VR experiences designed to simulate culturally diverse patient encounters [9]. Students found them authentic and engaging, and many reported feeling more comfortable communicating with people from other cultures. Similarly, Hunter et al. incorporated cultural immersion experiences into virtual simulations for dietetic students and discovered that participants felt more capable of responding to cultural diversity [10]. These resources expand access to virtually immersive experiences when in-person opportunities are less available.

Singh et al. explored allied health providers’ perspectives on cultural humility in palliative and end-of-life care. They found that working with culturally diverse patients fostered humility, ethical sensitivity, and reflection, but also revealed tensions between institutional protocols and patient or family values [11]. Similarly, Cáceres-Titos et al. reported that managing cultural diversity in end-of-life care frequently exposed ethical dilemmas and highlighted the need for providers to balance professional requirements with patient beliefs and rituals [12]. Taken together, these findings suggest that immersion and exposure experiences not only enhance interpersonal skills and humility but also illuminate the structural and ethical barriers that shape care delivery. Incorporating such lessons into curricula can prepare future clinicians to navigate these complexities with greater cultural responsiveness [11,12].

Immersion also reflects trends in interprofessional education. Brentnall et al. trialed a one-week simulation program for OT, PT, and podiatry students before placements [13]. Aiming for these higher-order learning outcomes, students highlighted strengths in communication, flexibility, and cultural competency during team-based immersion experiences that closely resembled the complexities of real-world practice. Overall, these studies demonstrate that immersion is a powerful tool for transformation, especially when enhanced by reflection, and when interprofessional teamwork and equitable participation are fundamental.

Coursework establishes a foundation in cultural constructs, health disparities, and communication techniques. Its success largely depends on pedagogy. Stough-Hunter et al. found that when students engaged in interactive discussions and case-based learning, they developed more advanced communication skills and a better understanding of disparities compared to those who only listened to a lecture [14]. Fryer et al. observed increased knowledge and awareness after a four-week module, but confidence in clinical application was lower, highlighting the potential limitations of theory without practical experience [15]. 

Arruzza and Chau conducted a systematic review of didactic interventions in allied health education, finding consistent improvements in knowledge and skills but variable effects on attitudes, with study quality differing across reports [16]. By contrast, Ozcan, Edeer, and Rust incorporated cultural content throughout PT and OT curricula using multiple methods: webinars, bias evaluations, reflective writing, and interprofessional panels [17]. Their longitudinal model showed significant gains from program start to graduation, indicating that culture is best delivered as an integrated curricular theme across courses and semesters. Emerging evidence highlights discipline-specific innovation. O’Fallon et al. introduced active learning into speech-language pathology training, combining case studies and discussions with guided reflection [18]. Student perceptions of their own self-awareness and readiness to serve bilingual and multicultural clients increased. This underscores the importance of designing didactic materials targeted to professional practice contexts rather than generic modules of broad application.

Technology can also serve to improve didactics. Fernández-Alcántara et al. examined digital simulation platforms that incorporate cultural and communicative training into the class curriculum [19]. These resources provided additional and conceptual reinforcement, as well as gave students practice navigating scenarios in a low-stress environment. Such strategies suggest that the boundary between didactic learning and applied learning is becoming more blurred.

Finally, issues with radiologic technology persist, and the number of LIMA programs is decreasing. Abrishami found modest increases in empathy after cultural blocks but reported strong student and faculty interest in more integration [20]. This aligns with a broader theme: when only extrinsic cultural elements are added, the results are limited. Didactic training is essential; however, it is most effective when engaging, targeted, and ongoing.

Training Workshops and Professional Development

Thirty-four workshops and seminars provide focused opportunities to raise awareness and practice skills. Chen et al. conducted a study that showed that PharmD students improved their critical cultural competence skills after participating in intensive cultural competence workshops. However, these skills diminished without ongoing practice and reinforcement [21]. Similar findings were reported by Jones and Pinto-Zapp; students became more aware of bias but lost momentum without clinical relevance [22].

Kempny et al. provided a partial list of online diversity training programs in health education [23]. In their review, participants showed consistent increases in knowledge and self-efficacy, as well as greater willingness to challenge prejudice. However, few studies tracked outcomes beyond the classroom, and questions about long-term effects remained. This aligns with previous workshop research: short-term gains are easy, but lasting impact depends on reinforcing efforts.

Workshops using simulated environments show promise in this development area. Walkowska et al. performed a meta-analysis of 27 simulation-based studies using SPs and found strong evidence for improvements in cultural competence, confidence, and learner satisfaction [24]. Walshe et al. also supported cultural simulations but warned against stereotyping if cases were too simple [25]. Careful design, a wide range of situations, and facilitated reflection are therefore essential.

Recent work adds nuance. Brentnall et al. demonstrated that immersive interprofessional workshops enhance students' readiness for complex team-based care, improving cultural skills and collaboration [13]. Chae et al. incorporated workshops into immersive VR environments, showing not only feasibility but also high learner engagement [9]. Both studies suggest that workshops do not have to be “one and done”; when presented in creative, practice-based formats, they gain more profound significance.

Finally, the success of the workshops depends on institutional backing. Without continuous faculty development and integration into the curriculum, workshops risk becoming isolated events. Including them as part of the required curriculum and professional development, rather than an optional addition, can help ensure they remain a central skill in allied health.

Synthesis

The body of evidence so far shows that using immersion, coursework, or workshops alone is not enough to prepare allied health students for delivering culturally competent care [3,4,7,8,13,16]. Immersion is powerful but can be expensive and logistically difficult [8,12]. Coursework provides an important conceptual foundation but can become too theoretical if not applied [6,15,16]. Workshops and simulations aim to develop skills and understanding, but they often end without a proper follow-up [14,17,19,25]. Research indicates that combining didactic coursework to build a foundation, immersion to enhance learning through real experiences, and workshops or simulations to offer repeated practice and feedback yields stronger and more sustainable results [7,10,13,19,23]. Reflection is also key; it ensures that even powerful immersive experiences have lasting effects [8,12].

Despite these strengths, many gaps remain in the research. Most studies are weak methodologically and rely on self-reports rather than objective outcomes, raising questions about whether skills are actually applied in practice [5,15,17]. Few longitudinal studies track whether students retain gains after graduation or whether these improvements translate to better patient care [7,23]. In addition, most interventions are small, context-specific, and not easily generalizable [14,16,25]. Some allied health professions such as speech-language pathology, radiologic sciences, respiratory therapy, and dietetics are notably underrepresented compared with nursing and physical therapy [11,20,18]. A lack of standardized assessment tools across studies further complicates efforts to compare results or establish benchmarks for success [6,21,22].

Curriculum-based programs that integrate cultural competence throughout training, rather than as isolated or optional courses, along with interprofessional learning and repeated experiential opportunities, tend to produce more consistent, longer-lasting gains [7,13,16,23]. Advances in virtual simulation, telehealth, and digital case-based learning offer promising opportunities for realistic and accessible training [9,10,19]. The review highlights an urgent need to address gaps related to measurement quality, long-term tracking, and inclusion of underrepresented allied health disciplines [20,22,23]. Ultimately, integrating cultural competence throughout allied health education enhances not only technical and clinical skills but also human qualities such as humility, empathy, and adaptability - qualities central to providing equitable, patient-centered care [1,2,11,12].

Limitations

This article has some limitations that need to be acknowledged. It was a narrative review and did not follow the systematic literature search or appraisal process of a systematic review or meta-analysis; as a result, some relevant studies might have been overlooked. The literature in this field is also quite varied, with differences in intervention duration, types, sample sizes, and measurement tools. This variability limits the ability to compare results across studies. In addition, most research relies on self-reported surveys or reflective activities that measure perceived growth, not sustainable behavioral change in professional practice or measurement validity.

Moreover, a large portion of the evidence comes from high-income countries, especially the U.S., which hampers the ability to generalize findings globally, given the diverse healthcare systems and cultural contexts. Overall, these limitations highlight the need for more rigorous, controlled, and longitudinal studies examining both short- and long-term effects of cultural competence training. Future research should prioritize validated multi-method assessments and consider various international perspectives to enhance the generalizability of findings and link educational outcomes to improvements in patient care.

## Conclusions

The literature shows that cultural competence learning in allied health is not about isolated or one-time instruction. Immersions offer an opportunity for transformative learning, allowing participants to experience cultural differences and reflect on their own biases. Lecture-based training provides the theoretical foundation in cultural competence, health disparities, and communication. Still, it needs to be complemented by experiential learning that bridges knowledge gained during coursework into practice. Workshops and simulations give concrete ways to expose students and the public to information that can increase awareness of risks and develop practical skills. However, their effectiveness diminishes without reinforcement or integration into the curriculum.

The most substantial evidence supports an integrated, longitudinal model of culturally competent care within the allied health curriculum. Programs that sequentially build structured immersion, coursework, and workshops across relevant educational stages lead to lasting improvements in student awareness, skills, and confidence. In addition, an interprofessional and simulation-based approach extends learning beyond the classroom, preparing students for interdisciplinary collaboration in modern healthcare settings.

Ultimately, cultivating cultural competence in allied health is both an educational priority and a matter of health equity. Developing and implementing broad-based, collaborative teaching strategies for allied health educators is essential to influence the progression of diverse health professionals toward meeting patients' needs. By adapting education to reflect changing demographics, educators can shape providers who are not only clinically competent but also empathetic, adaptable, and committed to addressing health disparities.
